# Guidance for manuscript submissions testing the use of generative AI for systematic review and meta-analysis

**DOI:** 10.1017/rsm.2025.10058

**Published:** 2025-12-11

**Authors:** Oluwaseun Farotimi, Adam Dunn, Caspar J. Van Lissa, Joshua Richard Polanin, Dimitris Mavridis, Terri D. Pigott

**Affiliations:** 1 University of Tennessee, Knoxville, USA; 2 Sydney School of Public Health, Faculty of Medicine and Health, The University of Sydney, Australia; 3 Methodology, Tilburg University, Tilburg School of Social and Behavioral Sciences, Netherlands; 4 University of San Francisco, USA; 5 Department of Primary Education, University of Ioannina, Greece; 6 College of Education and Human Development, Georgia State University, USA

Machine learning (ML) and generative artificial intelligence (GenAI) have great potential to improve key stages of systematic reviews and meta-analyses such as searching, screening (title/abstract and full-text), and data extraction. Research Synthesis Methods welcomes manuscripts that evaluate ML and GenAI methods across different stages of the systematic review and meta-analysis (SRMA) process. This guidance outlines requirements for manuscripts that evaluate the performance of ML or GenAI methods in SRMA, detailing expectations for the reporting of the methodology, validation, and results of these evaluations. Note that GenAI models differ from ML models in that the outputs of GenAI models can vary depending on the prompt, the model version, and random chance. Thus, evaluating the use cases of GenAI models in stages of SRMA requires particular attention. This guide adopts the principles itemized in other ongoing efforts, such as Responsible AI in Evidence Synthesis^1^ guidance and recommendations, and Digital Evidence Synthesis Tools^2^ to ensure responsible and transparent use of AI in SRMA methodologies.

## 1 Research design and method

Authors should clearly describe their research design. The experimental design must demonstrate a robust methodology that allows for replication and validation across different SRMA contexts. Thus, authors should detail (where applicable): (1) sampling methodology and dataset characteristics (specify whether the sample includes all studies or only a subset, with explicit reporting of both the number of all studies and subsets); (2) variables under consideration; (3) preprocessing methods; (4) clearly defined research questions; (5) heterogeneity considerations; (6) prompts (specify how prompt was developed and tested relative to the current study); and (7) methodological justification, including appropriate use cases, limitations, and scenarios when it may be inappropriate to use.

## 2 Evaluation

Evaluation of GenAI tools in SRMA must follow rigorous experimental design principles and incorporate established performance metrics. Authors must assess tool performance across different conditions to understand potential heterogeneity in a tool’s use across conditions, including sensitivity to different prompts, contexts, and data types.

Performance evaluation should utilize established metrics relevant to SRMA tasks. For example, if the purpose of the tool is to reduce human workload (workload reduction), the manuscript should report one or more metrics that reflect time or effort saved/spent, such as work saved over human screening, number needed to read, precision, accuracy, specificity, and sensitivity. While novel metrics may be proposed, they must complement, not replace, established metrics. Model performance should be benchmarked against traditional manual methods and/or existing automated approaches where available, with clear documentation of comparative advantages and limitations.

## 3 Validation

Authors must describe how AI/ML outputs were validated. Authors should conduct external validation to demonstrate their tool’s generalizability to sample set(s) independent from the training data. Comparative performance metrics should also be provided. If there is any risk of undisclosed training/test cross-contamination (e.g., because the testing data were available and may have been scraped as part of the training data for the model), this risk must be disclosed, and steps should be taken to estimate the model’s performance on data unlikely to have been part of the training data. Also, authors must describe their quality assurance procedures such as: (1) the oversight method employed (e.g., human-in-the-loop, automated verification, post-hoc sampling), (2) the extent of review (e.g., percentage of outputs, sampling strategy), (3) addressing errors, and (4) refinement processes.

## 4 Reproducibility and transparency

Authors must document specific random seed values for processes, including data splitting, model initialization, sampling procedures, and cross-validation steps. The documentation must detail the programming language, framework versions, and precise workflow points where randomization occurs. To ensure reproducibility, authors must provide complete analysis code and make all datasets (training, testing, and validation) openly accessible through established repositories, with clear documentation of their characteristics and context.

Authors must ensure full computational reproducibility by providing:

1. **Data availability:** All datasets (training, testing, and validation sets) must be made publicly accessible through established repositories (e.g., Zenodo, OSF, and DataVerse) with thorough documentation including appropriate metadata, dataset characteristics, license type, access procedures, and any usage restrictions.
2. **Code availability:** Complete, executable analysis code including preprocessing steps, analysis methods, and evaluation metrics.
3. **Random seed documentation:** Specify random seed values used during any of the following processes: data splitting, model initialization, sampling procedures, and cross-validation steps, and areas where randomization occurs.
4. **Computational environment:** Provide (1) hardware specifications (for local models) or API versions and access dates (for commercial models) and (2) any software dependencies with exact version numbers.
5. **Model specifications:** All model parameters, hyperparameters, and configurations necessary for replication.

## 5 Transparency and disclosure

Transparency demands candid disclosure of both technical and methodological aspects. Authors must include the following information in the manuscript:

1. Research methods following standard guidelines, including detailed data sources, algorithms, and validation procedures.
2. Explicit acknowledgement of model limitations, uncertainties, and potential biases, including those inherent in training data.
3. Mitigation strategies for identified biases.
4. All claims about model capabilities with supporting empirical evidence from the study; unsubstantiated assertions must be avoided.
5. All financial and non-financial interests, including funding sources and commercial relationships.

## Data Availability

Not applicable.
